# Impact of real-time ultrasound guidance on complications of percutaneous dilatational tracheostomy: a propensity score analysis

**DOI:** 10.1186/s13054-015-0924-7

**Published:** 2015-04-29

**Authors:** Venkatakrishna Rajajee, Craig A Williamson, Brady T West

**Affiliations:** Departments of Neurosurgery and Neurology, University of Michigan, Ann Arbor, MI 48109 USA; Survey Research Center, Institute for Social Research, Center for Statistical Consultation and Research, University of Michigan, Ann Arbor, MI 48109 USA

## Abstract

**Introduction:**

Recent studies have demonstrated the feasibility of real-time ultrasound guidance during percutaneous dilatational tracheostomy, including in patients with risk factors such as coagulopathy, cervical spine immobilization and morbid obesity. Use of real-time ultrasound guidance has been shown to improve the technical accuracy of percutaneous dilatational tracheostomy; however, it is unclear if there is an associated reduction in complications. Our objective was to determine whether the peri-procedural use of real-time ultrasound guidance is associated with a reduction in complications of percutaneous dilatational tracheostomy using a propensity score analysis.

**Methods:**

This study reviewed all percutaneous dilatational tracheostomies performed in an 8-year period in a neurocritical care unit. Percutaneous dilatational tracheostomies were typically performed by trainees under guidance of the attending intensivist. Bronchoscopic guidance was used for all procedures with addition of real-time ultrasound guidance at the discretion of the attending physician. Real-time ultrasound guidance was used to guide endotracheal tube withdrawal, guide tracheal puncture, identify guidewire entry level and confirm bilateral lung sliding. The primary outcome was a composite of previously defined complications including (among others) bleeding, infection, loss of airway, inability to complete procedure, need for revision, granuloma and early dislodgement. Propensity score analysis was used to ensure that the relationship of not using real-time ultrasound guidance with the probability of an adverse outcome was examined within groups of patients having similar covariate profiles. Covariates included were age, gender, body mass index, diagnosis, Acute Physiology and Chronic Health Evaluation II score, timing of tracheostomy, positive end-expiratory pressure and presence of risk factors including coagulopathy, cervical spine immobilization and prior tracheostomy.

**Results:**

A total of 200 patients underwent percutaneous dilatational tracheostomy during the specified period, and 107 received real-time ultrasound guidance. Risk factors for percutaneous dilatational tracheostomy were present in 63 (32%). There were nine complications in the group without real-time ultrasound guidance: bleeding (n = 4), need for revision related to inability to ventilate or dislodgement (n = 3) and symptomatic granuloma (n = 2). There was one complication in the real-time ultrasound guidance group (early dislodgement). The odds of having an adverse outcome for patients receiving real-time ultrasound guidance were significantly lower (odds ratio = 0.08; 95% confidence interval, 0.009 to 0.811; *P* = 0.032) than for those receiving a standard technique while holding the propensity score quartile fixed.

**Conclusions:**

The use of real-time ultrasound guidance during percutaneous dilatational tracheostomy was associated with a significant reduction in procedure-related complications.

## Introduction

Percutaneous dilatational tracheostomy (PDT) is a well established and widely utilized technique in the ICU, with a safety profile that compares favorably to surgical tracheostomy [1-3]. The reported incidence of significant complications for PDT is about 1 to 10%, including both short-term (such as bleeding, loss of airway and infection) and long-term (tracheal stenosis, tracheomalacia, tracheo-cutaneous fistula, and so forth) complications [1-3]. Many of these complications are potentially preventable and may be related to technical and procedural factors. Attention to the level of placement may have an impact on the risk of tracheal stenosis, trachea-innominate fistula and dislodgment [4-8]. Selection of the appropriate tube size and puncture site may decrease the risk of early dislodgement [9]. Avoidance of vascular structures may decrease the risk of bleeding [10-13]. Accurate assessment of endotracheal tube (ETT) tip position may decrease the risk of airway loss [14-16]. These procedural considerations may be particularly relevant in patients with high-risk factors, which may increase the technical difficulty of the procedure and the risk of complications. These high-risk factors include coagulopathy, morbid obesity, cervical spine immobilization (CSI), repeat tracheostomy and the ongoing need for high levels of respiratory support [17].

While bronchoscopic guidance is routinely used during PDT, bedside ultrasound has, more recently, received attention as a potentially useful tool to improve the safety of PDT. Pre-procedural assessment with ultrasound was described several years ago, as was the use of ultrasound during the procedure to facilitate tracheal puncture at the appropriate level, without real-time visualization of needle passage [7,9-12,18-20]. Our group has previously demonstrated the feasibility of real-time ultrasound guidance (RUSG) during PDT, including in patients with high-risk factors, with real-time visualization of tracheal puncture in the axial plane [21]. Several investigators have since demonstrated that RUSG-guided PDT (RUSG-PDT) is feasible and potentially useful [22-29]. The value of RUSG may be greatest in patients with high-risk factors, such as CSI and morbid obesity [19,20,23]. Two recent randomized controlled trials have demonstrated that the use of RUSG may significantly improve technical success and first-pass success rates [27,28]. While providing a compelling argument for the incorporation of RUSG into routine practice, these clinical trials were either not powered to detect a difference in complication rates or excluded patients with high-risk factors such as coagulopathy and CSI, who may be most likely to benefit from RUSG. No study, to our knowledge, has specifically evaluated the impact of RUSG on complication rates in a population with a significant proportion of high-risk factors.

Our objective therefore was to review all PDTs performed at our institution over an 8-year period, and determine the impact of RUSG on the risk of procedure-related complications. Given that this study was observational in nature and did not feature a randomized design, we employed a propensity score analysis to control for differences in risk factors and baseline variables which may have influenced the selection of a particular technique.

## Materials and methods

Approval of the University of Michigan Institutional Review Board was obtained for this study (HUM00062598). A waiver of informed consent was granted as the study involved analysis of data from an existing dataset and medical records. Data on all tracheostomies performed in the neurointensive care unit at the University of Michigan is prospectively entered into a database for the purposes of quality-assurance and ongoing review of complications. We reviewed information from the database, supplemented by information from the medical records, of all patients who underwent PDT in the neurointensive care unit of the University of Michigan between 2005 and 2013. Patients who underwent surgical tracheostomy were excluded. We confined our analysis to the neuroscience ICU because RUSG is predominantly used in the neuro-ICU at our institution. Restricting the analysis to neuro-ICU patients provided the additional benefit of relative homogeneity in the patient population, the types of illnesses and the individuals performing the procedures. Patients with less than 30 days of follow-up were excluded.

## Outcome of interest

The primary outcome of interest was the occurrence of a significant peri-procedural complication of PDT. The specific complications included in the composite outcome measure included both short- and long-term adverse events. Short-term complications included loss of airway during procedure, cardiac arrest during the procedure, bleeding requiring intervention, stomal or mediastinal infection, posterior wall injury, pneumothorax, pneumomediastinum, nerve injury, sustained hypoxia (>5 minutes) during the procedure, false passage of tube, inability to complete procedure, conversion to surgical tracheostomy, need for revision of tracheostomy, tracheal granuloma and early dislodgement (within 7 days). Delayed complications included tracheomalacia, tracheal stenosis, tracheoinnominate fistula and tracheo-esophageal or tracheo-cutaneous fistula/delayed closure. Bleeding requiring intervention included the need for any transfusion of blood products, electrocautery or other surgical hemostasis, revision of tracheostomy and discontinuation of medically necessary antithrombotic or antiplatelet therapy.

## Risk factors and other covariates

The medical record was reviewed for the presence of high-risk factors present at the time of PDT, which were then included in the propensity score analysis. These included the presence of coagulopathy, CSI, repeat tracheostomy, presence of thyroid mass over the trachea and inability to palpate the cricoid cartilage or visualize the first tracheal ring on ultrasound. Morbid obesity and high positive end-expiratory pressure (PEEP) requirement were also considered high risk factors, with body mass index (BMI) and PEEP at the time of procedure included as continuous variables in the propensity score analysis. Other variables in the propensity score analysis included age, gender, days from admission to tracheostomy, primary diagnosis category and Acute Physiology and Chronic Health Evaluation (APACHE) II score. The primary diagnosis categories were traumatic brain injury, subarachnoid hemorrhage, intracerebral hemorrhage, acute ischemic stroke, spinal cord injury, status epilepticus, brain tumor, and other. Coagulopathy was defined as the presence of a laboratory abnormality suggestive of impairment in coagulation and/or the use of therapeutic anticoagulation or intensive antiplatelet therapy. Laboratory abnormalities indicative of coagulopathy were: platelet count <50,000/uL, International Normalized Ratio >1.7, partial thromboplastin time (PTT) >1.5 times the normal value and/or fibrinogen <100 mg/dL. The abnormal laboratory result had to be present on the day of the procedure, without pre-procedural correction, to meet the definition of coagulopathy. Patients on unfractionated heparin and warfarin were considered coagulopathic only if the appropriate PTT and International Normalized Ratio criteria were met, or, in the case of unfractionated heparin, if the infusion was restarted and a therapeutic PTT recorded within 12 hours of completion of PDT. Anticoagulant agents included in the definition of coagulopathy, regardless of laboratory abnormality, included therapeutic use of low molecular weight heparin, direct thrombin inhibitors and direct Xa inhibitors. Anticoagulation with these agents, as well as with unfractionated heparin and warfarin, was typically discontinued or reversed the morning of the procedure, and had to be restarted within 12 hours of PDT to meet the definition of coagulopathy. Intensive antiplatelet therapy, defined as combination therapy with aspirin and clopidogrel, was not held or reversed for PDT.

## Timing and selection of technique

Timing of tracheostomy was decided on a case-by-case basis, with tracheostomy typically performed when the attending intensivist determined that the patient would likely require airway protection or mechanical ventilation for 3 weeks or longer. Selection of technique, including use of RUSG-PDT versus standard PDT (S-PDT), was at the discretion of the intensivist performing the procedure. Several intensivists staffing the unit during this period were not trained in performance of, or not actively performing, PDTs and therefore typically referred patients to the otolaryngology service for surgical tracheostomy. The baseline characteristics of patients referred for surgical tracheostomy (age, gender, days from admission to tracheostomy, BMI, PEEP, primary diagnosis category, APACHE II score and presence of the high-risk factors listed above) were compared to those of the PDT group to assess for a possible selection bias. All PDTs were typically performed by fellows or residents directly supervised by attending intensivists with at least 2 years experience with PDT and at least 1 year experience with RUSG, when used. All intensivists actively performing PDT during this period were trained by one of the authors (VR) in performance of RUSG-PDT. Bronchoscopy was utilized for all PDTs. The Ciaglia Blue Rhino® PDT kit (Cook Medical Inc, Bloomington, IN) was used for all patients. Tracheostomy tubes used included the Shiley™-DCT (sizes 6 and 8), Shiley™-XLT (sizes 6 and 7) (Covidien, Dublin, Ireland) and the Cook VersaTube™ (sizes 8 and 9) (Cook Medical Inc, Bloomington, IN). Selection of tracheostomy size and length was at the discretion of the intensivist performing the procedure, with selection typically based on patient height, gender, body habitus, neck anatomy and measurement of pretracheal soft tissue thickness when RUSG was used.

## Standard technique

A propofol infusion was typically utilized for sedation along with fentanyl for analgesia and a nondepolarizing agent, typically vecuronium, for neuromuscular blockade. The patient was positioned with the head in extension except when CSI was present, when the head was maintained in the neutral position. The ETT was withdrawn using bronchoscopic guidance and/or direct laryngoscopy until the tip was approximately at the level of the cricoid cartilage. An incision was first made following administration of local anesthetic (1% lidocaine with epinephrine), blunt dissection with a hemostat performed, then tracheal puncture performed under bronchoscopic guidance. The intent in all cases was to puncture the trachea between the first and the fourth rings in the anterior quadrant. The guidewire was then inserted and an initial stoma created with a 14 Fr dilator. This was followed by passage of the single-stage Ciaglia Blue Rhino® dilator over the guide catheter and the guidewire. The tracheostomy tube fitted on the appropriate loading dilator was then introduced into the trachea over the guide catheter and the guidewire and secured with sutures. Confirmation of tube position was with bronchoscopy and chest radiography.

## Technique of real-time ultrasound guided percutaneous dilatational tracheostomy

**Figure 1:**
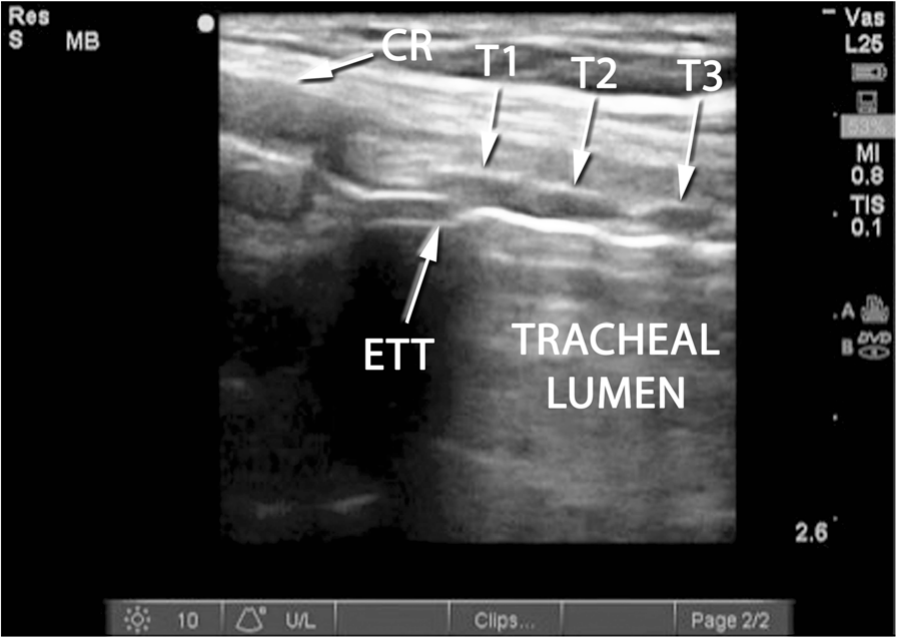
Endotracheal tube position on ultrasound. Longitudinal view of the trachea, demonstrating final positioning of the endotracheal tube (ETT) tip prior to tracheal puncture. CR, cricoid cartilage in cross section; T1, first tracheal ring in cross section; T2, second tracheal ring in cross section; T3, third tracheal ring in cross section.

**Figure 2:**
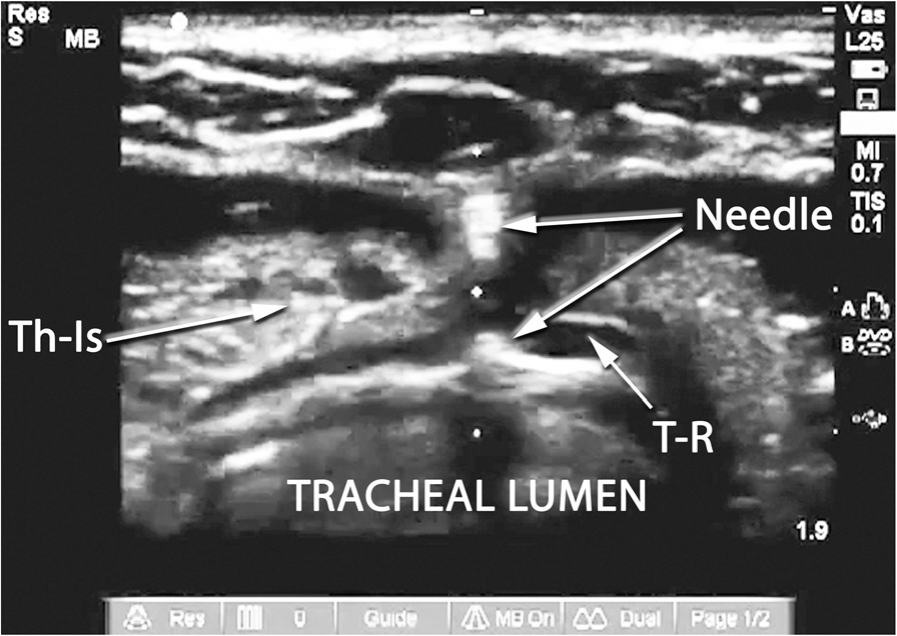
Anterior tracheal wall puncture on ultrasound. Axial view of the trachea demonstrating the visualized part of the needle at the anterior tracheal wall; Th-Is, thyroid Isthmus; T-R, tracheal Ring.

**Figure 3:**
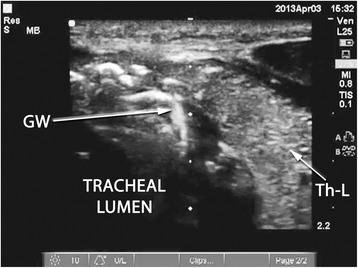
Confirmation of guidewire position (axial). Axial view of the trachea following guidewire (GW) passage, seen entering the tracheal lumen to the right of the midline. Th-L, thyroid lobe.

**Figure 4:**
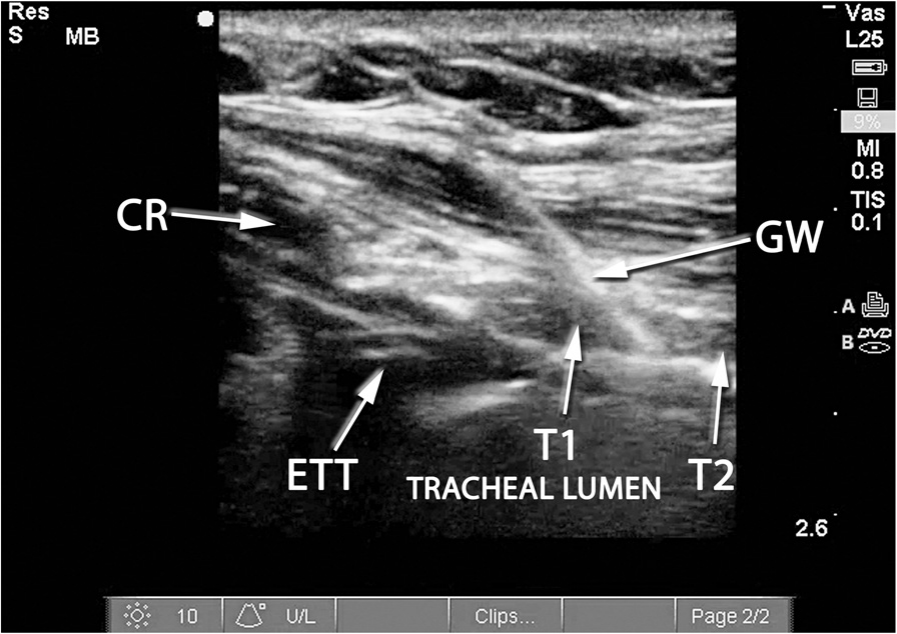
Confirmation of guidewire position (longitudinal). Longitudinal view of the trachea following guidewire (GW) passage, seen entering the trachea between the first and second tracheal rings. CR, cricoid cartilage in cross section; ETT, endotracheal tube tip; T1, first tracheal ring in cross section; T2, second tracheal ring in cross section.

**Figure 5:**
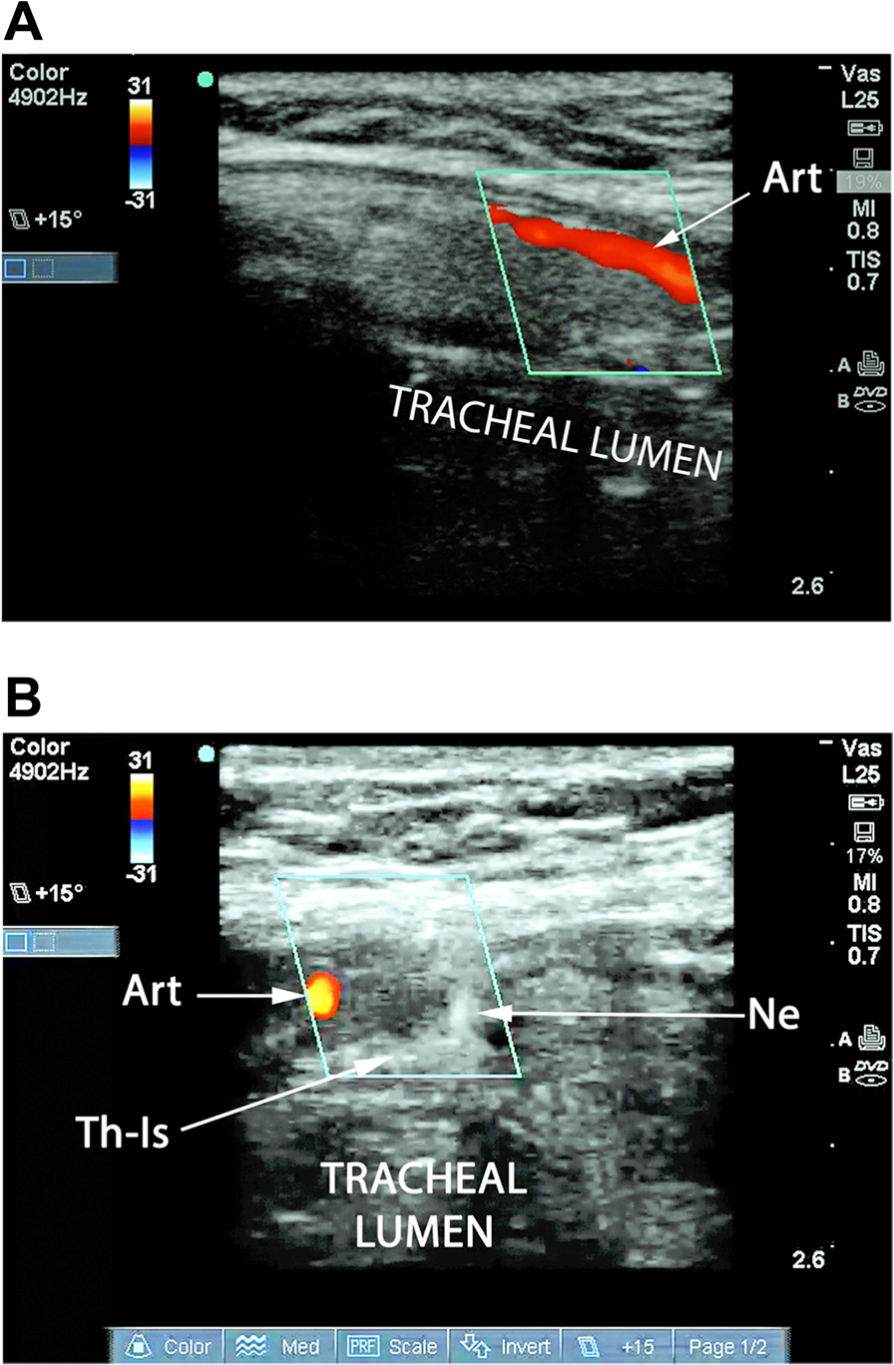
Visualization and avoidance of vascular structures. **(A)** Longitudinal view of the trachea with duplex imaging. A paramedian artery is seen, likely the thyroid ima. **(B)** Axial view of the trachea during puncture with duplex imaging (same patient as in (A)). The needle tip is directed to the anterior tracheal wall under real-time duplex guidance while avoiding the previously seen paramedian artery (likely the thyroid ima). Art, artery; Ne, needle tip; Th-Is, thyroid isthmus.

A Sonosite M-Turbo® (SonoSite Inc., Bothell, WA, USA) point-of-care ultrasound machine was used for all RUSG-PDTs, with either a 13–6 MHz or 10–5 MHz linear array probe within a sterile sheath. The RUSG-PDT technique differed from the standard technique in several ways. First, pre-procedural assessment of anatomy was performed with ultrasound, following appropriate patient positioning, to delineate vascular structures, pretracheal tissue thickness and intended site of puncture. Where a vascular structure was identified in the tracheostomy field, the patient was not automatically assigned to surgical tracheostomy. Instead, RUSG was used to avoid the vascular structure during tracheal puncture. Withdrawal of the ETT was performed under direct ultrasound guidance, using the technique previously described [16], until the tip was directly visualized under the cricoid cartilage (Figure 1). Where RUSG was used to withdraw the ETT, concurrent bronchoscopy/direct laryngoscopy was not performed while the tube was being withdrawn. Tracheal puncture was performed prior to incision of the neck, with the intent to enter the trachea between the first and fourth rings under RUSG. Selection of axial versus longitudinal (out-of-plane versus in-plane) view during puncture was at the discretion of the intensivist performing the procedure, although the axial view was utilized in most instances to facilitate puncture in the midline. The needle was then tracked from the skin to the anterior tracheal wall (Figure 2). Once the needle tip was directly visualized penetrating the anterior tracheal wall, the guidewire was introduced through the needle. Guidewire entry at the desired tracheal ring level and entry in the anterior quadrant were then confirmed in both the axial (Figure 3) and longitudinal (Figure 4) planes using ultrasound before skin incision and blunt dissection. Bronchoscopy was not utilized during RUSG tracheal puncture; instead, the bronchoscope was introduced following guidewire insertion to confirm intra-luminal passage. Following placement of the tracheostomy tube, lung sliding was assessed bilaterally over the anterior chest to confirm tube placement (in addition to bronchoscopic and radiographic confirmation) and evaluated for atelectasis (from blood or mucus occlusion of the bronchial passages) or pneumothorax [30]. Additional steps incorporating RUSG were utilized in patients with high-risk factors. Pre-procedural measurement of pre-tracheal tissue thickness with ultrasound was performed in patients with morbid obesity or an unusually thick neck to aid selection of tracheostomy tube length and size, as previously described [9]. The skin to second tracheal ring thickness was measured using ultrasound with the head in the neutral position. A tracheostomy tube with distance from flange to cuff (proximal plus radial length) at least 1.5 cm greater than this distance was then selected (for example, an extended proximal length Shiley™ 60XLT tube with proximal plus radial length of 6.1 cm versus a Shiley™ DCT-6 tube with proximal plus radial length of 3.8 cm). In patients with prior tracheostomy, RUSG was utilized to guide puncture at the site of prior tracheostomy to minimize additional scar tissue. In patients with coagulopathy, specific care was taken to avoid the following vascular structures during puncture: inferior thyroid veins, arterial branches along the isthmus and trachea (Figure 5A,B), the thyroid isthmus itself and any thyroid mass lesions. Axial and longitudinal views on ultrasound, in conjunction with varying angles of entry and levels of puncture, were utilized to guide the needle past these structures while attempting to enter the trachea between the first and fourth rings. The inferior thyroid veins and thyroid isthmus were not routinely avoided in patients without coagulopathy, however, and were punctured if necessary to obtain entry at the desired level. Of note, ultrasound-guided withdrawal of the ETT (versus withdrawal under bronchoscopic or laryngoscopic guidance) and measurement of pre-tracheal soft tissue thickness were performed variably at the attending physician’s discretion, while the other steps incorporating RUSG described above were performed for all patients undergoing RUSG-PDT.

## Statistical analysis

Descriptive statistics computed for the variables of interest included frequencies for categorical variables and median with interquartile range (IQR) for continuous variables. Bivariate associations of categorical variables with the outcomes of interest were tested using the Chi-square test or Fisher’s exact test, as appropriate. Bivariate associations of continuous variables with the binary outcomes of interest were assessed using the Mann–Whitney U test. Propensity score analysis was used to ensure that the relationship of not using ultrasound guidance with the probability of an adverse outcome was examined within groups of patients having similar covariate profiles. Propensity score analysis is useful in estimating the effect of an intervention (RUSG) in a retrospective observational study while adjusting for the covariates, or baseline variables, that predict receiving the intervention [31,32]. Propensity score analysis decreases the bias due to confounding variables that can occur when the intervention effect is estimated by simply comparing outcomes among patients receiving the intervention to those that did not. Logistic regression modeling was first used to predict the probability of receiving RUSG for each patient (that is, the propensity score) as a function of several potential confounding variables: age, gender, BMI, days from admission to tracheostomy, primary diagnosis category, APACHE II score, PEEP at time of procedure, presence of coagulopathy, presence of CSI, prior tracheostomy, presence of thyroid mass over the trachea and inability to palpate the cricoid cartilage or visualize the first tracheal ring on ultrasound. The predicted probabilities (propensity scores) were then recoded into quartiles, and a new factor was computed indicating the quartile to which each patient was assigned based on their propensity score. A logistic regression model was then fitted to the adverse outcome indicator (complication of tracheostomy), including the indicator of receiving RUSG and the propensity score quartile as independent variables. Inclusion of the propensity score quartile in this logistic regression model evaluating the predictive value of RUSG usage for occurrence of complications therefore helps account for the differential probability of receiving the intervention (RUSG) based on the covariate profile. The threshold for statistical significance was *P* < 0.05. All statistical analysis was performed using IBM SPSS Statistics for Macintosh, Version 22.0 (Armonk, NY: IBM Corp).

## Results

A total of 384 tracheostomies were performed in the neuro-ICU of the University of Michigan in the study period. Of these, 232 were PDT while the remainder were surgical. No single baseline variable demonstrated a statistically significant difference between patients undergoing PDT and surgical tracheostomy. Thirty-two patients who underwent PDT were excluded because of non-availability of at least 30 days of follow-up. Of note, none of these thirty-two excluded patients suffered a complication of tracheostomy during the limited period of follow-up available. Of 200 patients included in the analysis, 107 (53.5%) underwent RUSG-PDT while 93 (46.5%) underwent S-PDT. The distribution of variables in patients undergoing RUSG-PDT and S-PDT is shown in Table 1. A high-risk factor was present in 63 of 200 patients (31.5%): coagulopathy in 26 (13%), CSI in 25 (12.5%), PEEP requirement >10 cmH_2_O in 14 (7%), BMI >40 in 8 (4%) and prior tracheostomy in 2 (1%). The median duration of follow-up was 206 (IQR 653) days, median 197 (IQR 739) days in the S-PDT group and median 222 (IQR 551) days in the RUSG-PDT group (*P* = 0.31). No patient in either group was assigned to surgical tracheostomy following pre-procedural clinical/anatomical (in both groups) or pre-procedural ultrasound (in the RUSG group) examination. Inability to palpate the cricoid cartilage or visualize the first tracheal ring on ultrasound (or other unfavorable anatomic feature) was not specifically documented in any patient. Selection of tracheal tube length on the basis of sonographic measurement of pre-tracheal tissue thickness was documented in 28 patients (27% of the RUSG-PDT group), with an extended-length tube selected in 20 (71%) of these patients.

**Table 1:** **Distribution of variables in patients undergoing RUSG-PDT and S-PDT**

**Variable**	**S-PDT (n = 93)**	**RUSG-PDT (n = 107)**	***P*** **value from multivariate analysis**
Age in years (median (IQR))	58 (21)	54 (28)	0.11
Female gender (n (%))	39 (42%)	56 (52%)	0.29
Body mass index in kg/m^2^ (median (IQR))	27 (6)	27 (8)	0.55
Diagnosis (n (%))			
-Subarachnoid hemorrhage	27 (29%)	25 (23%)	0.14
-Traumatic brain injury	15 (16%)	14 (13%)	0.66
-Intracerebral hemorrhage	15 (16%)	20 (19%)	0.24
-Acute ischemic stroke	11 (12%)	15 (14%)	0.28
-Neuromuscular respiratory failure	6 (6%)	10 (9%)	0.30
-Spinal cord injury	5 (5%)	8 (7%)	0.33
-Status epilepticus	8 (9%)	5 (5%)	0.72
-Other	3 (3%)	7 (7%)	0.46
-Brain tumor	3 (3%)	3 (3%)	0.99
Days from admission to tracheostomy (median (IQR))	5 (6)	6 (6)	0.18
APACHE II score (median (IQR))	18 (7)	19 (10)	0.19
PEEP at time of procedure in cmH_2_O (median (IQR))	5 (0)	5 (3)	0.42
Cervical spine immobilized (n (%))	10 (11%)	15 (14%)	0.44
Coagulopathy (n (%))	12 (13%)	14 (13%)	0.79
Repeat tracheostomy (n (%))	0 (0%)	2 (2%)	0.99

Distribution of variables in patients undergoing real-time ultrasound guided percutaneous dilatational tracheostomy (RUSG-PDT) and standard percutaneous dilatational tracheostomy (S-PDT), with *P* value from multivariate analysis representing the *P* value from a logistic regression model predicting use of real-time ultrasound guidance. APACHE, Acute Physiology and Chronic Health Evaluation; IQR, interquartile range; PEEP, positive end-expiratory pressure.

## Complications

A significant complication was present in 10 of 200 patients (5%), and the list of significant complications is shown in Table 2. Nine of 93 patients (10%) in the S-PDT group and 1/103 (1%) patients in the RUSG group had a complication (*P* = 0.007). The single complication in the RUSG-PDT group was an early dislodgment (on day 6) in an agitated patient with cirrhosis and hepatic encephalopathy and previous tracheostomy. In the subgroup of patients without high-risk factors (n = 137), 5/67 (7%) patients in the S-PDT group and 0 of 70 (0%) in the RUSG-PDT group suffered a complication (*P* = 0.026). In the subgroup of patients with high-risk factors (n = 63), 4/26 (15%) patients in the S-PDT group versus 1/37 (3%) patients in the RUSG-PDT group suffered a complication (*P* = 0.15). Among 26 patients with coagulopathy, 4/12 (33%) who underwent S-PDT versus 0/14 who underwent RUSG-PDT suffered bleeding requiring intervention (*P* = 0.033).

**Table 2:** **Complications of percutaneous dilatational tracheostomy**

**Patient number**	**RUSG used**	**High-risk factor present**	**Nature of complication**	**Days from procedure**	**Details**	**Technical/procedural issue identified**
1	N	None	Bleeding requiring intervention, inability to complete procedure	0	Large neck hematoma during procedure, surgical tracheostomy and hemostatsis performed urgently in OR	Laceration of arterial branch along superior border of isthmus
2	N	Coagulopathy: dual antiplateley therapy	Bleeding requiring intervention	1	Copious persistent bleeding from stoma, requiring platelet transfusion and cessation of dual antiplatelet therapy	Vascular injury likely, specific source not identified
3	N	None	Tracheal granuloma	13	Inability to perform routine tube change at bedside, fiberoptic evaluation revealed a large tracheal granuloma causing luminal stenosis. Soft tracheal tube introduced over fiberoptic scope	None
4	N	None	Early dislodgment, need for surgical revision	5	Tracheal tube dislodged, inability to replace at bedside. Surgical revision required, initial stoma noted to be below 6th tracheal ring	Too low placement of tracheal tube
5	N	Coagulopathy: from therapeutic plasma exchange on consecutive days.	Bleeding requiring intervention	3	Persistent oozing with large hematoma in upper left quadrant of stoma. Surgical hemostasis with Surgicell® Fibrillar® absorbable hemostats	Vascular injury: focal bleeding identified at bedside at upper left quadrant of stoma
6	N	Coagulopathy: warfarin for venous thromboembolism	Death, bleeding requiring intervention	266	Massive bleeding and death from trachea-innominate fistula	Too low placement of tube on autopsy, proximity to innominate artery.
7	N	Coagulopathy: anticoagulation for venous thromboembolism	Bleeding requiring intervention	7	Persistent copious oozing during and after procedure. Anticoagulation reversed; surgical hemostasis performed in OR on day 7	Vascular injury: focus of bleeding identified in OR
8	N	None	Need for revision of tracheostomy	1	Persistent large air leak with loss of >30% tidal volume. Fiberoptic evaluation and emergent bedside revision performed post-procedure day 1	Too low placement: tube seen below 6th tracheal ring with suboptimal positioning on fiberoptic evaluation
9	N	None	Need for revision of tracheostomy, early dislodgment	4	Persistent large (20-25%) cuff leak post-procedure with dislodgment and inability to ventilate on day 4, surgical revision in OR	Too low placement of tracheal tube (below 8th ring) with consequent poor positioning
10	Y	Coagulopathy: end-stage liver disease, repeat tracheostomy	Early dislodgment	6	Tube dislodged following agitation and head shaking with subsequent brief period of hypoxia. Extended length tube replaced at bedside into stoma over a bougie.	? Inappropriate selection of tube length. Sonographic measurement pre-tracheal tissue thickness not performed.

Details of complications in the standard percutaneous dilatational tracheostomy and real-time ultrasound guidance (RUSG) percutaneous dilatational tracheostomy groups. N, no; OR, operating room; Y, yes.

## Propensity score analysis

No variable attained statistical significance in the logistic regression model constructed to predict the probability of receiving RUSG (Table 1), suggesting that the two groups of patients were fairly well balanced in terms of covariate profiles. In the logistic regression model fitted to the adverse outcome indicator (occurrence of a tracheostomy complication) including the indicator of receiving RUSG and the propensity score quartile, the odds of an adverse outcome for patients who underwent RUSG was found to be significantly lower (odds ratio = 0.087; 95% confidence interval, 0.009 to 0.811; Table 3) than among patients who received the standard technique, while holding the propensity score quartile fixed. The uncertainty associated with the estimated odds ratio reflects the small sample size and the small number of adverse outcomes (10/200). Despite these limitations, the coefficient for ultrasound receipt was still significantly different from zero (*P* = 0.032) when controlling for propensity score quartile.

**Table 3:** **Real-time ultrasound guidance as a predictor of any tracheostomy complications**

**Variables in the equation**	***P*** **value**	**Odds ratio**	**95% confidence interval**
Real-time ultrasound guidance	0.032	0.087	0.009-0.811
Propensity score quartile 1	0.891		
Propensity score quartile 2	0.716	0.616	0.045-8.402
Propensity score quartile 3	0.884	1.199	0.106-13.601
Propensity score quartile 4	0.900	1.172	0.100-13.753
Constant	0.053	0.109	

Logistic regression analysis of propensity score quartiles and use of real-time ultrasound guidance as predictors of any complications of tracheostomy.

## Discussion

Our study examined the impact of RUSG on the occurrence of complications following PDT, using propensity score analysis to account for any disparities in baseline variables that may have influenced selection of a particular technique. The use of RUSG was associated with a statistically significant 10-fold reduction in complications, while holding the propensity score quartile fixed. While several studies have demonstrated the feasibility and technical advantages of RUSG, our study is significant in, to our knowledge, being the first to demonstrate a clinically meaningful reduction in complications. Table 2, which describes the 10 complications seen in this study, may be particularly useful in demonstrating the value of RUSG. of the nine complications seen in the S-PDT group, at least seven had a specific technical factor identified which, plausibly, may have resulted in, or contributed to, the complication. In all these cases, the use of RUSG may have minimized the impact of these risk factors. This is particularly noticeable in coagulopathic patients undergoing PDT; four of 12 (33%) coagulopathic patients in the S-PDT group versus none of 14 coagulopathic patients in the RUSG-PDT group suffered bleeding requiring intervention (*P* = 0.033). Three of the four coagulopathic patients in the S-PDT group who suffered bleeding had a focal point of bleeding identified, suggesting a vascular source that might, potentially, have been avoided with RUSG. Figure 5 illustrates how a paramedian artery (likely the thyroid ima in the figure) identified on ultrasound can be avoided using real-time guidance. The other potentially important technical factor observed was low tracheostomy tube placement (below the fourth tracheal ring) seen in one patient who died following a trachea-innominate fistula and three others requiring revision of tracheostomy. Low placement has been identified as a possible risk factor for trachea-innominate fistula [4,5]. Also, it is possible that the longer segment of the tracheal tube within pre-tracheal tissue in patients with too low a stoma, which may result from excessive caudal angulation of the needle following skin puncture, may result in sub-optimal positioning of the distal segment of the tube within the trachea, increasing the risk of dislodgment and cuff leaks. Autopsy studies have demonstrated that sub-optimal level of the tube may occur despite the use of bronchoscopic guidance [7].

While our study was unable to determine the relative technical success of PDT (level of placement, passage in midline) in the RUSG-PDT and S-PDT groups because of the limitations of retrospective review of procedural documentation, two recent randomized controlled trials have demonstrated that incorporation of RUSG improves the technical success of PDT [27,28]. Rudas and colleagues demonstrated that the use of RUSG was more likely to result in a successful first pass (87% first pass success versus 58%; *P* = 0.028) as well as a puncture closer to the midline (mean deviation from midline 15 ± 3 versus 35 ± 5 degrees; *P* = 0.001) [27]. Yavuz and colleagues demonstrated that the use of RUSG resulted in significantly fewer patients requiring multiple attempts (4% versus 14%; *P* = 0.003), while slightly extending the duration of the procedure (24 versus 19 minutes, *P* = 0.001) [28]. Of note, both studies reported a trend toward lower complication rates with RUSG: 22% versus 37% (*P* = 0.24) in the Rudas study, which was not powered to detect a difference in complication rates, and 8% versus 15% (*P* = 0.054) in the Yavuz study, a near halving of complications which fell just short of statistical significance, despite the exclusion of patients with coagulopathy and CSI. We believe that the findings of these two studies, in conjunction with the findings of our study, strongly argue for the incorporation of RUSG into routine practice when performing PDT, particularly in patients with high-risk factors and at academic centers where the majority of procedures may be performed by trainees under supervision. Ultrasound has the advantage of being a non-invasive tool with little additional cost, where a point-of-care ultrasound machine is otherwise available. In our experience, trainees become familiar with the relevant sonographic anatomy relatively quickly. Some authors have suggested, on the basis of observational studies, that PDT can be performed safely with RUSG alone, without bronchoscopic guidance [25,29,33]. The primary arguments favoring the routine use of bronchoscopy may be greater real-time control of the airway and the ability to visualize the entire procedure within the trachea itself, to minimize the risk of posterior wall injury and false passage.

The primary limitation of our study is its retrospective nature, although data were primarily obtained from a prospective quality assurance database. Selection of technique (RUSG-PDT versus S-PDT) was at the discretion of the attending physician, not through random assignment. The use of a propensity score analysis, however, is likely to have mitigated the impact of any confounding through an imbalance in baseline variables and risk factors. In fact, no specific variable was seen to independently predict the use of RUSG in our study. It is possible that inter-operator variability may have influenced the risk of complications; however, procedures were performed by a wide variety of critical care trainees supervised by attending physicians in both groups, as is typical for most academic/teaching institutions. As previously mentioned, we were unable to compare the rates of technical accuracy in the RUSG-PDT and S-PDT groups because of inconsistency in procedural documentation, including the observed level of placement and insertion in the midline. For the same reason, we were unable to compare the time taken to complete S-PDT versus RUSG-PDT. The relatively large magnitude of reduction in complications seen with RUSG may, in part, be related to two study-specific factors. First, most of the procedures in this study were performed by trainees under supervision and, second, a relatively large proportion (32%) of patients had at least one high-risk factor identified. It is possible that more experienced operators and selection of patients without high-risk factors, particularly coagulopathy, may mitigate the risk of complications and therefore the potential benefit of RUSG. While the absolute number of high-risk patients in our study (n = 63) was insufficient for meaningful statistical analysis of the value of RUSG in this subgroup, it is useful to note that, of 37 patients with high-risk factors who underwent RUSG-PDT in our study, none suffered a complication related to the specific risk factor. The single complication in the RUSG group (early dislodgment during a period of agitation) was likely unrelated to the high-risk factors present in that patient (coagulopathy and previous tracheostomy). The population in this study was drawn from the neuro-ICU; therefore, the generalizability of its findings to patients in general medical/surgical ICUs is uncertain. In these units, the primary indication for tracheostomy is more likely to be refractory respiratory failure where gas exchange and hemodynamic stability may be more influenced by the technique and duration of the procedure, whereas in our study only 7% of patients required PEEP > 10 cmH_2_O at the time of the procedure. Where acute respiratory failure is a greater concern, the ability to minimize the occlusive effect of the bronchoscope in the airway, through primary use of RUSG for critical portions of the procedure such as ETT withdrawal and initial puncture, may, in fact, be more important.

The minimum duration of follow-up of 30 days may also have been insufficient to detect differences in the rates of long-term complications. The median duration of follow-up was over 6 months in both groups, however, with no statistically significant difference in median follow-up period between groups and only one long-term complication noted in the study (a delayed trachea-innominate fistula). Finally, the influence of unmeasured confounders cannot be entirely excluded.

## Conclusion

The use of RUSG during PDT was associated with a significantly lower rate of procedure-related complications in a propensity score matched analysis.

## Key messages

- The use of RUSG during PDT may decrease the risk of complications.
- RUSG may be particularly useful when performing PDT in patients with risk factors, such as coagulopathy.

## Abbreviations

APACHE: Acute Physiology and Chronic Health Evaluation
BMI: body mass index
CSI: cervical spine immobilization
ETT: endotracheal tube
IQR: interquartile range
PDT: percutaneous dilatational tracheostomy
PEEP: positive end-expiratory pressure
PTT: partial thromboplastin time
RUSG: real-time ultrasound guidance
RUSG-PDT: real-time ultrasound guided percutaneous dilatational tracheostomy
S-PDT: standard percutaneous dilatational tracheostomy

## References

[1] Delaney A, Bagshaw SM, Nalos M (2006). Percutaneous dilatational tracheostomy versus surgical tracheostomy in critically ill patients: a systematic review and meta-analysis. Crit Care.

[2] Barbetti JK, Nichol AD, Choate KR, Bailey MJ, Lee GA, Cooper DJ (2009). Prospective observational study of postoperative complications after percutaneous dilatational or surgical tracheostomy in critically ill patients. Crit Care Resusc.

[3] Carrer S, Basilico S, Rossi S, Bosu A, Bernorio S, Vaghi GM (2009). Outcomes of percutaneous tracheostomy. Minerva Anestesiol.

[4] Ivankovic AD, Thomsen S, Rottenbborg CC (1969). Fatal haemorrhage from the innominate artery after tracheostomy. Br J Anaesth.

[5] Arola MK, Inberg M, Sotarauta M, Vanttinen E (1979). Tracheo-arterial erosion complicating tracheostomy. Ann Chir Gynaecol.

[6] Sarper A, Ayten A, Eser I, Ozbudak O, Demircan A (2005). Tracheal stenosis after tracheostomy or intubation: review with special regard to cause and management. Tex Heart Inst J.

[7] Sustić A, Kovac D, Zgaljardić Z, Zupan Z, Krstulović B (2000). Ultrasound-guided percutaneous dilatational tracheostomy: a safe method to avoid cranial misplacement of the tracheostomy tube. Intensive Care Med.

[8] Raghuraman G, Rajan S, Marzouk JK, Mullhi D, Smith FG (2005). Is tracheal stenosis caused by percutaneous tracheostomy different from that by surgical tracheostomy?. Chest.

[9] Szeto C, Kost K, Hanley JA, Roy A, Christou N (2010). A simple method to predict pretracheal tissue thickness to prevent accidental decannulation in the obese. Otolaryngol Head Neck Surg.

[10] Kollig E, Heydenreich U, Roetman B, Hopf F, Muhr G (2000). Ultrasound and bronchoscopic controlled percutaneous tracheostomy on trauma ICU. Injury.

[11] Muhammad JK, Patton DW, Evans RM, Major E (1999). Percutaneous dilatational tracheostomy under ultrasound guidance. Br J Oral Maxillofac Surg.

[12] Flint AC, Midde R, Rao VA, Lasman TE, Ho PT. Bedside ultrasound screening for pretracheal vascular structures may minimize the risks of percutaneous dilatational tracheostomy. Neurocrit Care. 2009;11:372–6. doi: 10.1007/s12028-009-9259-z. Epub 2009 Aug 13. 10.1007/s12028-009-9259-z 19680824

[13] Muhammad JK, Major E, Wood A, Patton DW (2000). Percutaneous dilatational tracheostomy: haemorrhagic complications and the vascular anatomy of the anterior neck. A review based on 497 cases. Int J Oral Maxillofac Surg.

[14] Ambesh SP, Singh DK, Bose N (2001). Use of a bougie to prevent accidental dislodgment of endotracheal tube during bedside percutaneous dilatational tracheostomy. Anesth Analg.

[15] Tonui PM, Nish AD, Smith HL, Letendre PV, Portela DR (2014). Ultrasound imaging for endotracheal tube repositioning during percutaneous tracheostomy in a cadaver model: a potential teaching modality. Ochsner J.

[16] Rodríguez SJ, Esteves LE (2011). Real-time ultrasound-guided percutaneous dilatational tracheostomy. Crit Care.

[17] Al-Ansari MA, Hijazi MH (2006). Clinical review: percutaneous dilatational tracheostomy. Crit Care.

[18] Hatfield A, Bodenham A (1999). Portable ultrasonic scanning of the anterior neck before percutaneous dilatational tracheostomy. Anaesthesia.

[19] Sustić A, Zupan Z, Eskinja N, Dirlić A, Bajek G (1999). Ultrasonographically guided percutaneous dilatational tracheostomy after anterior cervical spine fixation. Acta Anaesthesiol Scand.

[20] Sustić A, Zupan Z, Antoncić I (2004). Ultrasound-guided percutaneous dilatational tracheostomy with laryngeal mask airway control in a morbidly obese patient. J Clin Anesth.

[21] Rajajee V, Fletcher JJ, Rochlen LR, Jacobs TL (2011). Real-time ultrasound-guided percutaneous dilatational tracheostomy: a feasibility study. Crit Care.

[22] Chacko J, Nikahat J, Gagan B, Umesh K, Ramanathan M (2012). Real-time ultrasound-guided percutaneous dilatational tracheostomy. Intensive Care Med.

[23] Guinot PG, Zogheib E, Petiot S, Marienne JP, Guerin AM, Monet P (2012). Ultrasound-guided percutaneous tracheostomy in critically ill obese patients. Crit Care.

[24] Rudas M, Seppelt I (2012). Safety and efficacy of ultrasonography before and during percutaneous dilatational tracheostomy in adult patients: a systematic review. Crit Care Resusc.

[25] Chacko J, Brar G, Kumar U, Mundlapudi B (2015). Real-time ultrasound guided percutaneous dilatational tracheostomy - with and without bronchoscopic control: an observational study. Minerva Anestesiol.

[26] Dinh VA, Farshidpanah S, Lu S, Stokes P, Chrissian A, Shah H (2014). Real-time sonographically guided percutaneous dilatational tracheostomy using a long-axis approach compared to the landmark technique. J Ultrasound Med.

[27] Rudas M, Seppelt I, Herkes R, Hislop R, Rajbhandari D, Weisbrodt L (2014). Traditional landmark versus ultrasound guided tracheal puncture during percutaneous dilatational tracheostomy in adult intensive care patients: a randomised controlled trial. Crit Care.

[28] Yavuz A, Yılmaz M, Göya C, Alimoglu E, Kabaalioglu A (2014). Advantages of US in percutaneous dilatational tracheostomy: randomized controlled trial and review of the literature. Radiology.

[29] Gobatto AL, Besen BA, Tierno PF, Mendes PV, Cadamuro F, Joelsons D (2015). Comparison between ultrasound- and bronchoscopy-guided percutaneous dilational tracheostomy in critically ill patients: a retrospective cohort study. J Crit Care.

[30] Lichtenstein D (2014). Lung ultrasound in the critically ill. Curr Opin Crit Care.

[31] Rosenbaum PR, Rubin DB (1983). The central role of the propensity score in observational studies for causal effects. Biometrika.

[32] Heinze G, Jüni P (2011). An overview of the objectives of and the approaches to propensity score analyses. Eur Heart J.

[33] Abdulla S, Conrad A, Vielhaber S, Eckhardt R, Abdulla W (2013). Should a percutaneous dilational tracheostomy be guided with a bronchoscope?. B-ENT.

